# Developing an Evaluation Index System for Service Capability of Internet Hospitals in China: Mixed Methods Study

**DOI:** 10.2196/72931

**Published:** 2025-07-25

**Authors:** Mingge Xia, Qi Liu, Li Ma, Jingyu Wen, Yan Xue, Hao Hu, Min Li, Hong Wei

**Affiliations:** 1 State Key Laboratory of Quality Research in Chinese Medicine, Institute of Chinese Medical Sciences, University of Macau, Taipa, Macao; 2 Department of Medical Insurance, Sichuan Academy of Medical Sciences and Sichuan Provincial People's Hospital, University of Electronic Science and Technology of China, 32# W Sec 2, 1 Ring Road, Chengdu, China, 86 17708130826; 3 Pudong Institute for Health Development, Shanghai, China; 4 Institute for Hospital Management, Tsinghua University, Shenzhen, China; 5 Department of Medical Administration, Sichuan Academy of Medical Sciences and Sichuan Provincial People's Hospital, University of Electronic Science and Technology of China, Chengdu, China; 6 Centre for Pharmaceutical Regulatory Sciences, University of Macau, Taipa, Macao; 7 Department of Public Health and Medicinal Administration, Faculty of Health Sciences, University of Macau, Taipa, Macao

**Keywords:** internet hospital, web-based medical service, digital health, telemedicine, telehealth, service capacity, hospital management, China

## Abstract

**Background:**

Rapid advancements in web-based technology have significantly transformed the health care landscape. In China, internet hospitals have emerged as vital components of the health care system. This rapid growth highlights the necessity for a thorough evaluation of internet hospitals within the health care system, as they operate under models distinct from traditional health care settings.

**Objective:**

This study aimed to identify critical indicators that reflect the service capabilities of internet hospitals and to establish a comprehensive evaluation index system for their assessment.

**Methods:**

This study initially compiled a pool of indicators through literature review and expert consultation. The final evaluation index system was established using the Delphi method, involving 2 rounds of expert consultation, and the index weights were determined using the Analytic Hierarchy Process.

**Results:**

In total, 21 experts from relevant fields, such as hospital management, clinical services, and health information management, were enrolled in the consultation. After 2 rounds of Delphi consultation, the experts’ positive coefficients were 95.45% and 100%, and the authoritative coefficients were both >0.7. The final evaluation index system for the service capabilities of internet hospitals contained 3 first-level indicators, 9 second-level indicators, and 29 third-level indicators. The first-level indicators were categorized into 3 dimensions: “Internet hospital infrastructure,” “Internet hospital services,” and “Internet hospital management.” “Internet hospital infrastructure” encompasses the essential conditions for service delivery, such as hardware and software resources, human capital, information security, and payment systems. “Internet hospital services” focuses on the scope and depth of services offered, such as “online medical services,” “online pharmaceutical services,” and “collaborative services.” Finally, “Internet hospital management” is divided into “medical administration” and “general management.” Weights were assigned to each indicator using the Analytic Hierarchy Process, revealing that “Internet hospital services” held the highest importance (0.573) among 3 first-level indicators, followed by “Internet hospital infrastructure” (0.239) and “Internet hospital management” (0.188). Among the second-level indicators, “Online medical service” emerged as the most critical (0.344), followed by “Medical administration” (0.140), “Online pharmaceutical service” (0.119), “Collaboration service” (0.110), and “Information security” (0.087). Among the third-level indicators, “Online health consultation” (0.092) had the highest weight, followed by “Online chronic disease management” (0.080), “Online pharmaceutical consultation” (0.076), “Consistency between online medical service and offline medical service” (0.071), and “Medical quality management” (0.071).

**Conclusions:**

This study identified and established a comprehensive evaluation index system for assessing the service capabilities of internet hospitals in China. The resulting index system not only provides a valuable tool for evaluating and improving service delivery in internet hospitals but also serves as a foundation for future studies in this rapidly evolving field.

## Introduction

The rapid advancement of web-based technology has transformed the health care industry. In China, internet hospitals have emerged as vital components of the health care system [1,2]. Internet hospitals are medical platforms that combine online and offline access to medical institutions to directly provide patients with various online and telemedicine services, including online consultation, online diagnosis, follow-up treatment, and health management [3]. In July 2015, the State Council of China issued the Opinion on Actively Pushing Forward the Development of “Internet+” Action, which encouraged the development of online medical service platforms and emphasized the key role of Internet hospitals in providing online medical services [4]. In 2018, the National Health Commission (NHC) of China issued 3 foundational documents: Measures for the Administration of Internet Diagnosis and Treatment (Trial Implementation), Measures for the Administration of Internet Hospitals (Trial Implementation), and Specification for Telemedicine Service Management (Trial Implementation), establishing the legal framework for internet hospitals and online medical services [5]. This marked the first time that China introduced detailed regulations for internet hospitals, indicating a transition to a standardized development stage.

Driven by policy support and growing public acceptance of web-based health services, the number of internet hospitals in China has increased exponentially in recent years. By the end of 2024, China had >3000 internet hospitals, a dramatic increase from 26 in 2018 [6,7]. This rapid growth emphasizes the necessity for a thorough evaluation of internet hospitals within the health care system, as they operate in models distinct from traditional health care settings.

Alongside this expansion, studies on internet hospitals have increased, although most studies tend to focus on operational models and specific service components [8,9]. In assessing internet hospitals, Tao et al [10] used the SERVQUAL model to evaluate service quality and patient satisfaction in China. The SERVQUAL questionnaire encompasses 5 service quality dimensions. Zhang et al [11] constructed an evaluation index system for service function quality and technical quality for internet hospitals using a literature review and the Delphi approach. The index system consists of 9 second-level indicators, including safety, reliability, and assurance, and 29 third-level indicators. Li and Guo [12] constructed a set of online medical service quality indicators for public hospitals in China from the perspective of online and offline integration. The primary indicators and their weights are outcome quality (0.34), process quality (0.26), structure quality (0.22), and integration quality (0.18). Despite the rapid expansion of internet hospitals, existing evaluation frameworks primarily assess service quality and patient satisfaction. However, little attention has been given to their service capacity—an essential factor in ensuring sustainable health care delivery [13].

A hospital’s service capacity refers to its ability to provide medical services effectively, considering factors such as physical, human resource, technological, and operational capacity [14]. Understanding service capacity enables hospitals to meet patient demands more effectively, reduce wait times, and improve care quality. By assessing capacity, hospitals can pinpoint areas where resources are either underutilized or overburdened, allowing for a more efficient allocation of staff, equipment, and space [15]. Since 2014, China has gradually introduced standards and evaluation guidelines to assess the service capabilities of secondary and tertiary hospitals; however, these are limited to traditional offline hospitals [10]. In 2016, the NHC issued guidelines for evaluating the service capabilities of tertiary general hospitals. These guidelines encourage tertiary hospitals to enhance their overall capacity by highlighting 5 key aspects of service capability: resource allocation, human resources, diagnostic and treatment capabilities, work efficiency, and medical technology [16]. In 2019, the NHC issued guidelines for evaluating the service capabilities of townships and community health service centers, establishing standards for assessing the service capabilities of primary health care institutions [17]. The guidelines were updated in 2023 and fall into the following 4 areas of focus: functional tasks and resource allocation, primary medical and public health services, medical management, and general administration [18].

As an emerging concept, internet hospitals are experiencing rapid growth. However, they also face several challenges, including poor integration with the existing health care system, insufficient support mechanisms, and a lack of diverse development models. Therefore, a comprehensive understanding and scientific evaluation of their service capabilities are crucial for identifying issues and shortcomings, ultimately fostering improvements in service quality and supporting sustainable growth. Moreover, established standards for service capabilities can guide internet hospitals in strategic service planning and development tailored to their specific needs [19,20]. Given that the operating models of internet hospitals differ from those of traditional health care settings, how to accurately capture and evaluate their unique service capabilities is a crucial question. To address this gap, this study aimed to identify critical indicators that reflect the service capabilities of internet hospitals and to establish a comprehensive evaluation index system for their assessment. The findings of this study are expected to provide a valuable tool for evaluating and improving service delivery in internet hospitals and serve as a foundation for future studies in this rapidly evolving field.

## Methods

### Study Design

**Figure 1.**
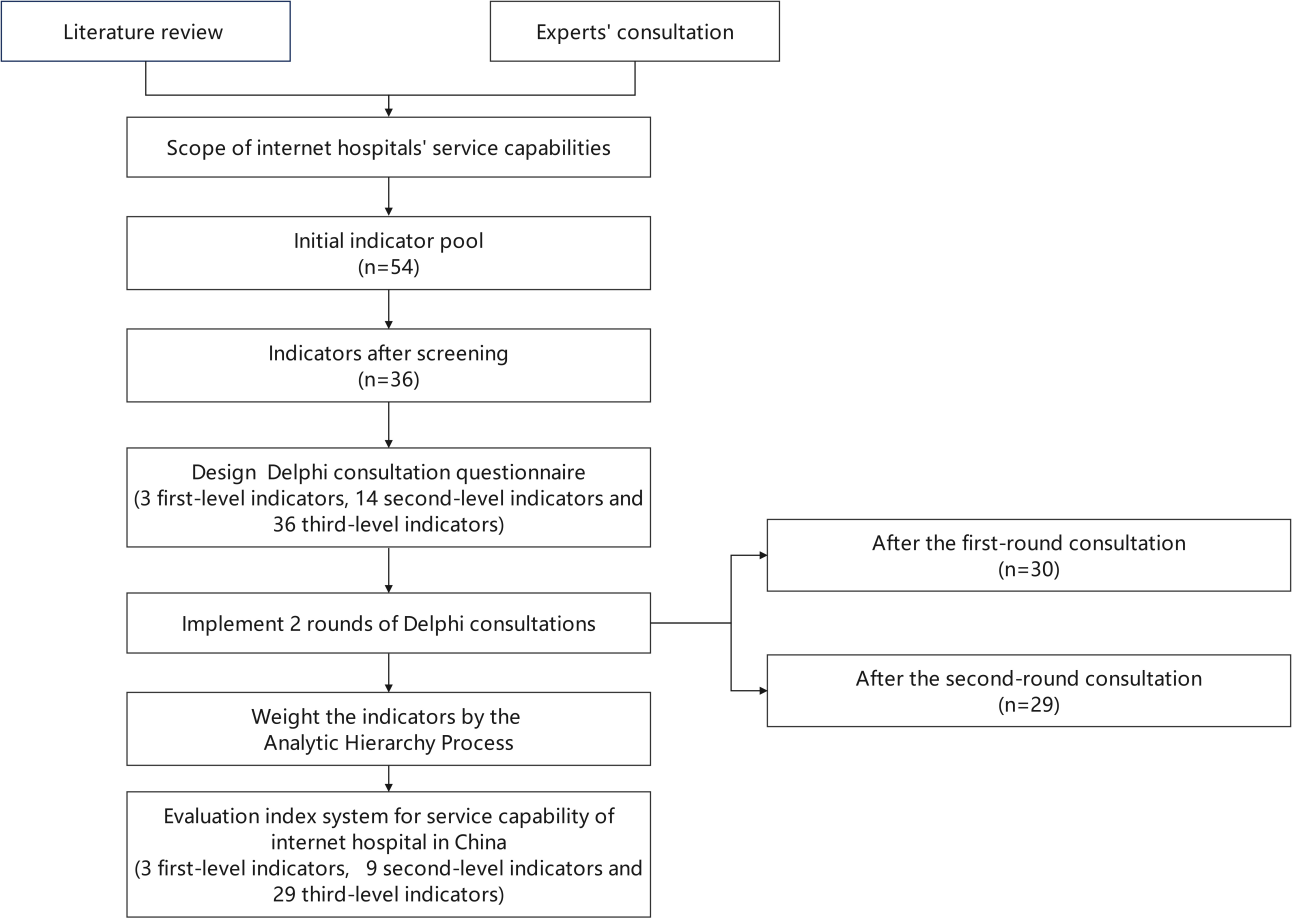
The research process.

This study first constructed an initial indicator pool based on literature research and expert consultations. The final evaluation index system was determined using the Delphi method through 2 rounds of expert consultation, and the weights of the indices were established using the Analytic Hierarchy Process (AHP). Figure 1 illustrates the research process used in this study.

### Construct Initial Indicator Pool

The initial indicator pool was constructed based on a literature review and expert consultation. We searched the PubMed, China National Knowledge Infrastructure, and Wanfang databases to gather potential indicators and descriptions published up to January 2024. Search terms included “internet hospital,” “telemedicine,” “mobile health,” “online health,” “remote medicine,” “service capacity,” “hospital capacity,” “health service capacity,” and “hospital evaluation.” The detailed search strategy is shown in Multimedia Appendix 1. The exclusion criteria were as follows: (1) commentaries, study protocols, letters, editorial material, and conference abstracts or posters; (2) studies unrelated to the evaluation of internet hospitals or web-based medical services; and (3) studies not using the English or Chinese language. Literature selection was conducted independently by 2 researchers (MGX and QL). Any disagreements were resolved through discussion and, if necessary, with the involvement of a third researcher (HW). After excluding duplicates, 199 papers remained, and 21 papers were considered based on the team members’ screening.

In addition, policy documents issued by the Chinese government regarding web-based medicine, internet hospitals, telemedicine, hospital capacity, and hospital evaluation were reviewed. The official websites for policy document searches included the State Council, People’s Republic of China, NHC, National Healthcare Security Administration, and the Sichuan Provincial Health Commission. Multimedia Appendix 2 provides the policy documents under consideration. We consulted 2 experts to discuss the importance and validity of the indicators based on data accessibility and feasibility. They did not participate in the Delphi method. Finally, 54 indicators were included in the initial pool.

### Inclusion Criteria of Experts

The inclusion criteria for Delphi consultation experts were as follows: (1) relevant work experience and familiarity with the research field, including internet hospital management and operations, online clinical services, health information management, and web-based medicine policy; (2) more than 5 years of professional experience; (3) holding a professional title of intermediate level or above; and (4) voluntary participation in the survey, with sufficient time and energy to complete the entire process.

### Delphi Process

The Delphi method is a structured communication technique used to gather expert opinions and reach a consensus. In health care, the Delphi method is widely used to achieve consensus on various topics, such as clinical guidelines, health policy decisions, and the development of quality indicators [21]. A Delphi expert consultation was conducted to screen and determine the final indicators.

Consultation questionnaires were designed to collect expert opinions based on the initial indicator pool, with an explanatory statement of the survey. The questionnaires mainly consisted of four parts: (1) experts’ basic information; (2) experts’ evaluation of the initial indicators (experts score the importance and feasibility of each indicator using a 5-point Likert scale: 5 means very important, 4 means important, 3 means moderately important, 2 means of little importance, and 1 means unimportant); (3) experts’ familiarity with each indicator (Cs), rated by the experts themselves (Cs was categorized as follows: extremely familiar [1.00 point], very familiar [0.80 points], generally familiar [0.60 points], less familiar [0.40 points], and unfamiliar [0.20 points]); and (4) expert’s judgment criteria (Ca), ranging from 1 to 3 (1 = low, 2 = medium, and 3 = high), to clarify the impact of theoretical analysis, practical experience, knowledge of literature, and intuitive feeling [22]. Quantization Table S1 is shown in Multimedia Appendix 3. In addition, a free-text box was provided to collect expert suggestions on modifications or deletions for each initial indicator. To manage disagreements, summary statistics and anonymized qualitative feedback from previous rounds were provided, allowing experts to reflect on differing viewpoints and reconsider their responses in light of group trends.

Two rounds of Delphi consultations were conducted between June 2024 and October 2024. Questionnaires were distributed online and offline. A professional online survey tool (WJX.cn) was used for the online distribution of the questionnaire. In the first round, each indicator was defined in detail and expert comments were collected. In the second round, we summarized the results and comments of the first round and modified or deleted any inappropriate indicators. After confirming the final indicators, we invited the experts who participated in the second round to complete the comparison matrix.

### Indicator Inclusion Criterion

The mean value of the importance score, full score frequency (percentage of experts with full marks), and coefficient of variation were used to determine the final evaluation index system [23]. Based on previous studies, the inclusion criteria for indicators using the critical value method were defined as follows: indicators with a mean importance score below 3.5, a coefficient of variation of >20%, or a full score frequency below 20%, was deleted or adjusted based on expert opinion [24]. The adjustments were summarized and sent to the experts for review. The final evaluation index system was confirmed until all experts had the same opinion.

### Weight Assignment of Each Indicator

Weighting criteria are crucial for developing an evaluation index system framework. Weights represent the “trade-offs” or “exchange rates” between criteria, allowing individual criterion value scores to be converted to a common scale [25]. AHP is a powerful tool in multiple-criteria decision analysis that helps define weights and establish a hierarchical structure for criteria within the framework [26]. It was developed by Saaty [27] and is one of the most well-known and widely used approaches for multiple-criteria decision-making. In this study, AHP was applied to assign weights to each indicator after finalizing the index system. Experts applied the Saaty 1‐9 scale to assess the relative importance of each pair of indicators, indicating both which indicator was more important and the degree of difference. The scale ranged from “equal importance” (1 point) to “absolute importance” (9 points), with intermediate descriptors, such as “moderate” (3 points), “strong” (5 points), and “very strong” (7 points). Even-numbered values (2, 4, 6, and 8) were used to express more nuanced preferences. Subsequently, the percentage weighting method was used to calculate the weights of the tertiary indicators. Specifically, within each secondary category, the weight of a tertiary indicator was determined by dividing its individual importance score by the total score of all tertiary indicators in that category and then multiplying the result by the weight of the corresponding secondary indicator [28]. We calculated the initial and combination weights of each indicator and conducted consistency testing using SPSSAU. When the consistency ratio (CR) is <0.10, the judgment matrix is considered to have satisfactory consistency; if the CR is >0.10, the judgment matrix must be adjusted accordingly.

### Data Analysis

Excel was used for data entry, and SPSS was used for data analysis. The authority coefficients (Cr) of the experts are the arithmetic average of the experts’ judgment criteria (Ca) and the degree of the experts’ familiarity (Cs). We calculated the Cr value based on the self-evaluation scores. A Cr value of ≥0.7 is considered to indicate acceptable reliability. The higher the Cr value, the higher the prediction accuracy. The degree of coordination of the experts’ opinions is expressed using Kendall coefficient of concordance (Kendall W). Generally, the value of Kendall W is between 0 and 1, and a greater Kendall W coefficient indicates better agreement between the experts’ estimates. *P* value of <.05 is considered statistically significant.

### Ethical Considerations

This study did not involve the health data of individuals, and ethics approval was not required according to the ethical review of the ethics committee of the Sichuan Provincial People’s Hospital. Sensitive data were not collected for this study, and no compensation was provided for participants. Online informed consent was obtained from all participants before they completed the questionnaires. All data relevant to this study will be stored on a password-encrypted computer, and only the researchers will have access to the data.

## Results

### Characteristics of Experts

In this study, we conducted 2 rounds of expert consultations and enrolled 21 experts from various provinces in China, including Sichuan, Shanghai, Chongqing, Beijing, Henan, and Shanxi. During the first-round consultation, 22 questionnaires were distributed, with a recovery rate of 95.45% (21/22). During the second-round consultation, we distributed 20 questionnaires to experts who completed the first-round survey, with a 100% recovery rate (20/20). Most experts in consultation have a master’s degree or above and are mainly specialized in hospital management, clinical service, and health information management. All experts had work experience in internet hospitals or health services. The demographic characteristics of the experts are summarized in Table 1.

**Table 1.** The characteristics of the Delphi participants.

Participants’ information	Values, n (%)	
Occupation		
Hospital management	7 (33.33)	
Clinical service	5 (23.81)	
Health information management	5 (23.81)	
Health authority	2 (9.52)	
Web-based technology	2 (9.52)	
Seniority (years)		
6‐10	6 (28.57)	
11‐15	3 (14.29)	
16‐20	7 (33.33)	
>20	5 (23.81)	
Professional title		
Middle	10 (47.62)	
Associate senior	9 (42.86)	
Senior	2 (9.52)	
Education		
Bachelor’s degree	8 (38.10)	
Master’s degree	12 (57.14)	
Doctor’s degree	1 (4.76)	

### Authority Coefficient and Degree of Coordination

The Cr value of the first-round expert consultation was 0.82 (Ca=0.88, Cs=0.75), and that of the second-round expert consultation was 0.90 (Ca=0.94, Cs=0.85), indicating that the expert consultation results were accurate and credible. The results of the Kendall W test for the indicators are shown in Table 2. Because the number of first-level indices was <7, only the second-level and third-level indices were analyzed. Kendall W values were 0.183 and 0.210, respectively, indicating an improvement over the first round. The differences were statistically significant according to the chi-square test (*P*<.001), suggesting that the degree of coordination among the experts was acceptable.

**Table 2.** The result of expert opinions’ coordination degree.

Hierarchical level	Kendall W	Chi-square (*df*)	*P* value
First round			
First level	0.041	N/A^a^	N/A
Second level	0.154	42.083 (13)	<.001
Third level	0.140	103.076 (35)	<.001
Second round			
First level	0.141	N/A	N/A
Second level	0.183	29.336 (8)	<.001
Third level	0.208	120.824 (29)	<.001

a Not applicable.

### Indicator Screening

According to the indicator inclusion criteria and expert opinions, in the first-round consultation, the research team modified 1 second-level indicator, merging 9 second-level indicators into 4. For the third-level indicator, the research team deleted 6 indicators, modified 9, merged 6 indicators into 3, and added 1. During the second-round consultation, the research team deleted 1 third-level indicator and modified 1 third-level indicator. Thus, the evaluation index system for the service capability of internet hospitals in China was confirmed, which included 3 first-level indicators, 9 second-level indicators, and 29 third-level indicators (Table 3).

**Table 3.** The evaluation index system for service capability of internet hospitals in China.

First-level indicators	Second-level indicators	Weights	Third-level indicators	Weights
1: Internet hospital infrastructure (0.239)	1.1: Hardware and software equipment	0.051	1.1.1: Hardware equipment	0.012
			1.1.2: Internet support	0.027
			1.1.3: Supervisory control and data acquisition	0.012
	1.2: Human resource	0.068	1.2.1: Number of physicians offering OMS^a^	0.024
			1.2.2: Number of pharmacists offering OMS	0.014
			1.2.3: Number of nurses offering OMS	0.012
			1.2.4: Number of technical and administration service personnel	0.008
			1.2.5: Proportion of senior title health professional offering OMS	0.011
	1.3: Information security	0.087	1.3.1: Information security protection	0.051
			1.3.2: Information use supervision	0.036
	1.4: Payment and reimbursement	0.032	1.4.1: Mobile payment	0.018
			1.4.2: Online settlement of medical insurance	0.014
2: Internet hospital service (0.573)	2.1: Online medical service	0.344	2.1.1: Online health consultation	0.092
			2.1.2: Online chronic disease management	0.080
			2.1.3: Remote medical consultation with specialists	0.050
			2.1.4: Remote medical monitoring	0.043
			2.1.5: Home care service	0.035
			2.1.6: Disease coverage of OMS	0.043
	2.2: Online pharmaceutical service	0.119	2.2.1: Online pharmaceutical consultation	0.076
			2.2.2: Medication delivery	0.043
	2.3: Collaboration service	0.110	2.3.1: Consistency between OMS and offline medical service	0.071
			2.3.2: Collaboration among institutions	0.039
3: Internet hospital management (0.188)	3.1: Medical administration	0.140	3.1.1: Medical quality management	0.071
			3.1.2: Pharmaceutical management	0.029
			3.1.3: Prevention and handling of medical disputes	0.040
	3.2: General management	0.049	3.2.1: Basic regulation or regulatory compliance	0.012
			3.2.2: Human resource management	0.011
			3.2.3: Performance management	0.014
			3.2.4: Emergency management	0.012

a OMS: Online medical service.

### Final Indicator Weights

The normalized and combination weights of each indicator were calculated using the AHP (Table 3). A higher weight indicates that the indicator was more important for the assessment. The CR of each modified judgment matrix was <0.10, indicating that all the judgment matrices had good consistency (Multimedia Appendix 4). For the first-level indicators, “Internet hospital infrastructure,” “Internet hospital service,” and “Internet hospital management,” the weight values were 0.239, 0.573, and 0.188, respectively. Among the second-level indicators, “Online medical service” (0.344) had the highest weight, followed by “Medical administration” (0.140), “Online pharmaceutical service” (0.119), “Collaboration service” (0.110), and “Information security” (0.087). Among the third-level indicators, “Online health consultation” (0.092) had the highest weight, followed by “Online chronic disease management” (0.080), “Online pharmaceutical consultation” (0.076), “Consistency between online medical service and offline medical service” (0.071), and “Medical quality management” (0.071).

## Discussion

### Principal Findings

This study aimed to identify key indicators that reflect the service capabilities of internet hospitals in China. Using a systematic approach involving a literature review and expert consultation, we initially developed a pool of potential indicators. The final evaluation index system was refined through 2 rounds of expert consultation to ensure a comprehensive representation of the diverse aspects of internet hospital service capacity. Finally, we established an evaluation index system for internet hospital service capabilities in China, consisting of 3 first-level indicators, 9 second-level indicators, and 29 third-level indicators.

The first-level indicators were categorized into 3 dimensions: “Internet hospital infrastructure,” “Internet hospital services,” and “Internet hospital management.” “Internet hospital infrastructure” encompasses the essential conditions for service delivery, such as hardware and software resources, human capital, information security, and payment systems. “Internet hospital services” focuses on the scope and depth of services offered, such as “online medical services,” “online pharmaceutical services,” and “collaborative services.” Within “online medical services,” this includes offerings, such as “online health consultations,” “online chronic disease management,” “remote consultations with specialists,” and “home care services.” Finally, “Internet hospital management” is divided into “medical administration” and “general management,” covering third-level indicators, such as “regulatory compliance,” “medical quality management,” and “performance management.”

Weights were assigned to each indicator using the AHP, revealing that “Internet hospital service” held the highest importance (0.573) among 3 first-level indicators, followed by “Internet hospital infrastructure” (0.239) and “Internet hospital management” (0.188). Among the second-level indicators, “Online medical service” emerged as the most critical (0.344), followed by “Medical administration” (0.140), “Online pharmaceutical service” (0.119), “Collaboration service” (0.110), and “Information security” (0.087). Among the third-level indicators, “Online health consultation” (0.092) had the highest weight, followed by “Online chronic disease management” (0.080), “Online pharmaceutical consultation” (0.076), “Consistency between online medical service and offline medical service” (0.071), and “Medical quality management” (0.071).

### Implications for Practice, Research, and Policy

The web is rapidly transforming people’s lives and permeating every sector, including health care. In the health industry, web-based medical services, such as telemedicine, mobile health (mHealth), and digital health, have emerged with technological advancements. These innovative service models help overcome geographical and time-related barriers, allowing patients to access health care quickly and conveniently. With their growing adoption, research evaluating online medical services and internet hospitals has also increased.

Akter et al [29] developed and validated a multidimensional, hierarchical service quality scale for assessing mHealth service quality. Their study revealed that consumers evaluate mHealth service quality based on 3 core dimensions: system, interaction, and information qualities [29]. Similarly, Powell et al [30] conducted semistructured, in-depth interviews with adult patients following online medical consultations and identified 5 key dimensions relevant for assessing telemedicine services: convenience, efficiency, communication, privacy, and comfort. Bashir and Bastola [31] evaluated the quality of telehealth nursing services using a modified SERVQUAL instrument, finding high overall satisfaction among respondents. Meanwhile, Rahman et al [32] designed a 10-item, 5-point Likert-scale questionnaire to assess patients’ experiences with virtual clinic visits across five domains: (1) patient satisfaction; (2) ease of use; (3) effectiveness, including improved access to care; (4) reliability; and (5) perceived usefulness. Their findings suggest that virtual clinic visits significantly save time and are considered beneficial by most participants [32].

Our study focuses on the service capacity of internet hospitals, and the results indicate that “Internet hospital services” and “Internet hospital infrastructure” were the key elements for evaluating the service capabilities of internet hospitals. Among these, the scope and depth of online medical services have emerged as the most critical. Currently, traditional medical models and web-based medicine are converging, with the scope of online medical services continuously expanding [2]. The model is evolving from a basic online consultation format to a more comprehensive approach, where online consultations form the foundation, complemented by video interactions, online diagnosis, and treatment processes from consultation to prescription and medication retrieval [8]. For instance, the Internet Hospital of Guangdong Traditional Chinese Medicine Hospital introduced an online chronic disease management service, allowing patients with chronic diseases to follow up and prescribe medicines at home with health insurance reimbursements [2]. Furthermore, innovative services, such as remote medical monitoring and telesurgical guidance, are exploring 5G networks and mixed-reality technologies [33]. To enhance service capabilities, internet hospitals must, on one hand, expand the range of medical services to offer comprehensive options, such as online consultations, remote health management, and home care. However, they must deepen their service offerings by innovating medical service models, such as developing web-based, full-course management for specialized conditions and providing patients with integrated, sustainable online and offline health care services. This approach will ultimately improve the efficiency and effectiveness of health care delivery.

In terms of hospital infrastructure, both physical and internet hospitals require a solid foundation to support their service capability. The NHC has established key standards for personnel, equipment, facilities, and medical specialties in internet hospitals, as outlined in the Measures for the Administration of Internet Hospitals [34-36]. For example, the guidelines specify that internet hospitals must have high-speed, reliable network access with a minimum bandwidth of 10 Mbps for business use, provided by at least 2 broadband providers. Additionally, internet hospitals are encouraged to use dedicated web-based lines and virtual private networks to ensure the quality of medical data transmissions. Beyond hardware, internet hospitals must also maintain stable and reliable technical support, including network operations, system maintenance, and data backup and recovery, to ensure the continuous availability and reliability of medical services.

Information security is also a crucial aspect of internet hospitals’ service capabilities. In compliance with relevant national laws and regulations, internet hospital information systems must implement level 3 information security protection [35]. Given the highly sensitive nature of patient medical data, internet hospitals must rigorously adhere to laws governing information security and data confidentiality, ensuring proper protection of patient information and preventing data breaches. Consequently, internet hospitals must also prioritize managing information usage and implementing effective oversight to maintain information security throughout the service process, thereby enhancing overall service capabilities.

Another important consideration is the unification of online and offline health care services. Traditional health care systems often struggle with accessibility and efficiency, particularly in rural and underprivileged areas. Internet hospitals have emerged as a potential solution for bridging the gap between online and offline health care services by creating a unified system that enhances patient access and convenience [37]. By integrating digital platforms with traditional in-person care, patients can receive timely consultation, diagnosis, and follow-up care on the web, while also having the option to visit physical health care facilities for more complex treatments or emergencies. This integration not only optimizes health care delivery by reducing wait times and improving accessibility but also ensures continuity of care, allowing for better coordination between online consultations and offline treatments [33]. Therefore, further development of internet hospitals should focus on integration with offline health care services to provide comprehensive, patient-centered care that adapts to evolving health care needs.

Moreover, policy support for internet hospitals should be further strengthened, particularly in the area of reimbursement. Currently, most services provided by internet hospitals in China are not covered by medical insurance, which significantly limits the utilization of web-based health care services [38]. Adequate reimbursement policies can help reduce financial barriers for patients, making web-based health care services more affordable and accessible, particularly for those in remote or underserved areas who may benefit the most from web-based health care. Moreover, a more predictable financial environment can encourage internet hospitals to innovate and expand their services. This can lead to better technologies, improved health care delivery, and more specialized services. Implementing the evaluation index system in real-world settings may present several challenges, necessitating both strategic planning and practical operational tools. In China, the widespread use of nonuniform IT systems across hospitals hinders the consistent extraction and reporting of evaluation indicators. To address this, establishing a multilevel governance structure is essential, one that involves health authorities, hospital management, IT vendors, and insurance payers. For instance, the Guangzhou Health Commission has developed a city-level health data exchange center that automatically collects data on outpatient volume, prescription patterns, and response times from all licensed internet hospitals [38]. Furthermore, pilot implementations in selected hospitals should be considered, as a standardized index system may not fully account for the variability in service delivery models or patient demographics. Finally, continuous training and capacity building are essential to ensure accurate implementation, as low awareness or misinterpretation of indicator definitions can lead to reporting errors.

### Strengths and Limitations

The innovative aspect of this study lies in the establishment of a comprehensive evaluation index system that effectively represents the service capabilities of internet hospitals in China. Using the Delphi method and AHP, we identified key indicators that were previously lacking in the literature. By identifying key indicators of service capacity, our study provides valuable insights for policy makers and health care administrators aiming to enhance the effectiveness and efficiency of internet hospitals. Specifically, the established evaluation index can guide the strategic planning and resource allocation necessary to improve service delivery, thereby addressing the growing demand for telehealth services in China. As the number of internet hospitals continues to increase, ensuring their operational readiness and capacity to deliver high-quality care will be paramount to achieving long-term health outcomes and reducing inequalities in health care access.

However, this study has some limitations. Reliance on expert opinions through the Delphi method, while robust in achieving consensus, may introduce subjective biases that could influence the selection and weighting of indicators. Additionally, the expert sample, although diverse, may not fully represent all relevant stakeholders involved in web-based health care service delivery. Future studies should aim to validate the proposed evaluation index across a broader range of internet hospitals and consider incorporating patient feedback and outcomes into the assessment process. Furthermore, longitudinal studies can provide deeper insights into how service capacities evolve over time and their impact on patient outcomes, thereby refining our understanding of internet hospitals in China’s dynamic health care system.

### Conclusions

This study successfully identified and established a comprehensive multidimensional evaluation index system for assessing the service capabilities of internet hospitals in China. Using the Delphi method and AHP, we developed a robust framework that included 3 first-level indicators, 9 second-level indicators, and 29 third-level indicators reflecting the service capability of internet hospitals. The resulting index system not only provides a valuable tool for evaluating and improving service delivery in internet hospitals but also serves as a foundation for future studies in this rapidly evolving field. As the health care environment continues to transform, ongoing efforts to adapt and enhance this index will be essential to ensure that internet hospitals can meet the growing demands of patients and effectively contribute to China’s health care system.

## Supplementary material

10.2196/72931 Multimedia Appendix 1 Search strategy.

10.2196/72931 Multimedia Appendix 2 Policy documents related to internet hospital and web-based medical service in China.

10.2196/72931 Multimedia Appendix 3 Quantization table of expert’s judgment criteria.

10.2196/72931 Multimedia Appendix 4 The result of consistency test of each judgment matrix.

## Abbreviations

AHP: Analytic Hierarchy Process
CR: consistency ratio
mHealth: mobile health
NHC: National Health Commission
