# Supplementary material for: Developing an Evaluation Index System for Service Capability of Internet Hospitals in China: Mixed Methods Study
Source: J Med Internet Res. 2025 Jul 25;27:e72931. doi: 10.2196/72931 (PMC12296255; doi:10.2196/72931)
Supplement: Multimedia Appendix 1 [file jmir-v27-e72931-s001.docx]

**Search strategy:**

PubMed:

("internet hospital"[Title/Abstract] OR "telemedicine"[Title/Abstract] OR "mobile health"[Title/Abstract] OR "online health"[Title/Abstract] OR "remote medicine"[Title/Abstract]) AND ("service capacity"[Title/Abstract] OR "service capability"[Title/Abstract] OR "hospital capacity"[Title/Abstract] OR "hospital capability"[Title/Abstract] OR "health service capacity"[Title/Abstract] OR "health service capability"[Title/Abstract] OR "hospital evaluation"[Title/Abstract] OR "capacity"[Title] OR "capability"[Title] OR "hospital assessment"[Title/Abstract])

China National Knowledge Infrastructure:

TKA=("互联网医院"+"互联网医疗"+"远程医疗") AND TKA=("服务能力") AND TKA=("评价")

Wanfang database:

题名或关键词:("互联网医院"or"互联网医疗"or"远程医疗") AND 题名或关键词:("服务能力"or"评价")

Language limitations:

1.English

2.Chinese

Date limitations:

Published before January 2024.
