# Supplementary material for: Developing an Evaluation Index System for Service Capability of Internet Hospitals in China: Mixed Methods Study
Source: J Med Internet Res. 2025 Jul 25;27:e72931. doi: 10.2196/72931 (PMC12296255; doi:10.2196/72931)
Supplement: Multimedia Appendix 2 [file jmir-v27-e72931-s002.docx]

**Policy documents related to internet hospital and internet medical service in China**

**中国互联网医院相关政策条例及通知公告**

| 发布时间  Publish time | 机构  Institution | 名称  Document names | 主要内容  Main contents |
| --- | --- | --- | --- |
| 2009.03 | 中共中央国务院  CPC Central Committee and the State Council | 《关于深化医药卫生体制改革的意见》  *Opinions of the CPC Central Committee and the State Council on Deepening the Reform of the Medical and Health Care System* | 较早推动医疗数字化建设的政策  To promote the construction of digital health |
| 2009.07 | 原卫生部  Ministry of Health | 《互联网医疗保健信息服务管理办法》  *Administrative Measures for Internet Medical and Health Information Services* | 提出推动互联网医疗信息服务的发展  To promote the development of Internet medical and health information services |
| 2013.09 | 国务院  The State Council | 《关于促进健康服务业发展的若干意见》  *Opinions of the State Council on Promoting the Development of the Health Service Industry* | 提出推进院内信息化与数据共享、在线挂号与咨询、物联网与智能设备的研发  To improve the hospital information systems, and online registration and consultation platforms. |
| 2014.08 | 原卫计委  National Health and Family Planning Commission | 《关于推进医疗机构远程医疗服务的意见》  *Opinions of National Health and Family Planning Commission on promoting telemedicine service in medical institutions* | 积极推动远程医疗服务发展  To develop telemedicine service |
| 2015.07 | 国务院办公厅  General Office of the State Council | 《关于积极推进“互联网+”行动的指导意见》  *Guidance of the State Council on promoting "Internet +" action* | 对“互联网+医疗”明确说明，对移动医疗/远程医疗/互联网诊疗等给出具体指导意见  To define the direction of "Internet +" in the medical field. To promote the new model of online health care. To support the development of medical information sharing service platform by the third-party institutions. |
| 2015.03 | 国务院办公厅  General Office of the State Council | 《全国医疗卫生服务体系规划纲要(2015-2020)》  *Planning of the General Office of the State Council on the national medical and health service system (2015-2020)* | 要发展健康中国、推动健康信息服务和智慧医疗服务  To clear promote the development of mobile Internet and telemedicine services. To promote health information service and smart medical service benefiting the whole people by information technology. |
| 2016.06 | 国务院办公厅  General Office of the State Council | 《关于促进和规范医疗大数据应用发展的指导意见》  *Guidance of the State Council on promoting and standardizing the application and development of healthcare big data* | 要发展医疗信息化、远程医疗，推动医疗大数据建设工作  To clear the development goals of healthcare big data, including the overall goals and specific goals, and emphasize its application and innovation. |
| 2016.10 | 中共中央国务院  The CPC Central Committee, the State Council | “健康中国2030”规划纲要  *Healthy China 2030 Plan* | 首次将健康医疗产业的发展提升到国家战略层面，明确“互联网+医疗”态度  To integrate "Internet + Medical Care and Health" into the national strategic planning |
| 2018.04 | 国务院办公厅  General Office of the State Council | 《关于促进“互联网+医疗健康”发展的意见》  *State Council's opinion about promoting the development of "Internet + Medical Care and Health"* | 健全“互联网+医疗健康”服务体系，鼓励医疗机构构建覆盖线上线下一体化医疗服务模式  To define the development direction, service system, supporting system, supervision system and safeguard system of "Internet + Medical care and Health". |
| 2018.07 | 国家卫健委  National Health Commission | 《关于深入开展“互联网+医疗健康”便民惠民活动的通知》  *Notice to further develop "Internet + Medical Care and Health" to benefit the people* | 加快推进智慧医院建设，运用互联网信息技术，改造优化诊疗流程，贯通诊前、诊中、诊后各环节  To develop "Internet + Medical Care and Health" services from 10 aspects: medical treatment, settlement payment, patient medication, public health, family doctor, telemedicine, health information, emergency treatment, government affairs sharing and inspection and inspection. |
| 2018.07 | 国家卫健委、国家中医药管理局  National Health Commission, National Administration of Traditional Chinese Medicine | 《互联网诊疗管理办法(试行)》  *Internet Medical Consultation Administrative Measures (Trial)* | 为互联网医院赋予合法的主体身份、提供监管准则，强调“实体医疗机构”在互联网医院中的核心地位  To formulate the administrative measures of Internet medical consultation and Internet hospital. To formulate the administrative standards of telemedicine services. To define the access, practice rules and supervision measures of Internet hospital. |
|  |  | 《互联网医院管理办法(试行)》  *Internet Hospital Administrative Measures (Trial)* |  |
|  |  | 《远程医疗服务管理规范(试行)》  *Telemedicine Services Administrative Standards (Trial)* |  |
| 2018.11 | 四川省人民政府办公厅  General Office of the People's Government of Sichuan Province | 《关于促进“互联网+医疗健康”发展的实施意见》  *Opinions on Promoting the Development of "Internet plus Medical Care and Health"* | 提供智能导医分诊、候诊提醒、检验检查结果查询、移动支付等线上服务  Propose to offer online services such as AI medical consultation, waiting list reminder, examination result inquiry, and mobile payment. |
| 2019.03 | 国家卫生健康委  National Health Commission | 《医院智慧服务分级评估标准体系（试行）》  *Smart Service Scoring System for Hospital Assessment (Trial)* | 医院智能服务项目的的分级评估标准  To establish the Smart Service Scoring System (4s) for hospital assessment |
| 2019.08 | 全国人民代表大会  National People's Congress | 新修订的《中华人民共和国药品管理法》  *Law of the People's Republic of China on Pharmaceutical Administration* | 删除不得通过网络直接销售处方药  To allow the online sales of prescription drugs from the legislative level. |
| 2019.09 | 国家医疗保障局  National Healthcare Security Administration | 《关于完善“互联网＋”医疗服务价格和医保支付政策的指导意见》  Guidance of the National Healthcare Security Administration on improving the "Internet +" medical service price and healthcare insurance payment policy | 明确“互联网+”医疗服务的医保支付政策  To clear the policy guidance for the healthcare insurance settlement of "Internet +" medical services. |
| 2019.09 | 四川省卫健委  Health Commission of Sichuan Province | 《四川省智慧医院评审标准（试行）》  Evaluation Standards for Smart Hospitals in Sichuan Province | 评审细则包括智慧医院基础、智慧医疗服务、智慧医院管理、信息标准应用、新型技术应用五个方面  The evaluation criteria cover five aspects: smart hospital infrastructure, smart medical services, smart hospital management, application of information standards, and application of new technologies. |
| 2020.02 | 国家卫健委  National Health Commission | 《关于在疫情防控中做好互联网诊疗咨询服务工作的通知》  *Notice on Promoting Internet medical consultation services in epidemic prevention and control* | 要充分发挥互联网诊疗咨询服务在疫情防控中的作用，加强对互联网诊疗服务的监管  To strengthen Internet medical consultation to support epidemic prevention and control |
| 2020.03 | 国家医保局  National Healthcare Security Administration | 《关于推进新冠肺炎疫情防控期间开展“互联网+”医保服务的指导意见》  *Guidance of advancing the "Internet +" healthcare insurance service in Novel coronavirus pneumonia prevention and control period* | 明确推进定点零售药店配药直接结算，互联网医保正式落地  To define the scope of healthcare insurance payment for internet health service. To encourage continuous online prescriptions. To allow online payment and direct settlement. |
| 2020.03 | 中共中央国务院  The CPC Central Committee and the State Council | 《关于深化医疗保障制度改革的意见》  *Opinions of the CPC Central Committee and the State Council on Deepening the Reform of Medical Insurance System* | 支持“互联网＋医疗”等服务模式创新发展，电子处方、诊疗费和药费医保在线直接结算  To support the innovative development of service models such as 'Internet + Healthcare,' and enable online direct settlement of electronic prescriptions, medical service fees, and drug costs through medical insurance |
| 2020.04 | 国家发展改革委  The National Development and Reform Commission | 《关于推进“上云用数赋智”行动培育新经济发展实施方案》  *The Implementation Plan for Promoting the Actions of “Migrating to Cloud, Using Digital Tools and Enabling Intelligence” and Fostering the Development of New Economy* | 探索推进互联网医疗医保首诊制和预约分诊制  To explore and promote the implementation of a first-visit system and an appointment-based triage system for web-based healthcare and health insurance services. |
| 2021.01 | 国家卫健委  National Health Commission | 《医疗机构设置规划指导原则（2021-2025年）》  *Guiding Principles for the Planning of Medical Institution Establishment (2021–2025)* | 大力发展互联网诊疗服务，将互联网医院纳入医疗机构设置规划  To promote the development of web-based diagnosis and treatment services, and incorporate internet hospitals into the planning of medical institution establishment |
| 2021.10 | 国家卫建委、中医药管理局  National Health Commission, National Administration of Traditional Chinese Medicine | 《公立医院高质量发展促进行动（2021-2025年）》  *Action for Promoting High-Quality Development of Public Hospitals (2021-2025)* | 对智慧医院建设及管理做出要求  To set requirements for the construction and management of smart and internet hospitals |
| 2022.03 | 四川省卫健委  Health Commission of Sichuan Province | 《四川省公立医院高质量发展促进行动（2022-2025年）》  *Action for Promoting High-Quality Development of Public Hospitals in Sichuan Province (2022-2025)* | 对互联网医院建设目标做出规划  To make plans for the construction goals of the Internet hospital |
| 2023.03 | 国务院办公厅  General Office of the State Council | 《关于进一步完善医疗卫生服务体系的意见》  *Opinions on Further Improving the Medical and Health Service System* | 完善“互联网+”医疗服务，发展“互联网+医疗健康”  To improve “Internet +” medical services and promote the development of “Internet + Healthcare. |
