# Supplementary material for: Developing an Evaluation Index System for Service Capability of Internet Hospitals in China: Mixed Methods Study
Source: J Med Internet Res. 2025 Jul 25;27:e72931. doi: 10.2196/72931 (PMC12296255; doi:10.2196/72931)
Supplement: Multimedia Appendix 3 [file jmir-v27-e72931-s003.docx]

Table S1. Quantization table of expert’s judgment criteria.

|  | Degree of influence | | |
| --- | --- | --- | --- |
| Judgment basis | High | Medium | Low |
| Theoretical analysis | 0.3 | 0.2 | 0.1 |
| Practical experience | 0.5 | 0.4 | 0.3 |
| Knowledge from domestic and foreign counterparts | 0.1 | 0.1 | 0.1 |
| Intuition | 0.1 | 0.1 | 0.1 |
