# Supplementary material for: Developing an Evaluation Index System for Service Capability of Internet Hospitals in China: Mixed Methods Study
Source: J Med Internet Res. 2025 Jul 25;27:e72931. doi: 10.2196/72931 (PMC12296255; doi:10.2196/72931)
Supplement: Multimedia Appendix 4 [file jmir-v27-e72931-s004.docx]

Table S1. The result of consistency test of each judgment matrix.

|  | λmax | C.L. | C.R. |
| --- | --- | --- | --- |
| 1-3 | 3.003 | 0.001 | 0.003 |
| 1.1-1.4 | 4.114 | 0.038 | 0.043 |
| 2.1-2.3 | 3.076 | 0.038 | 0.073 |
| 3.1-3.2 | 2 | / | / |
| 1.1.1-1.1.3 | 3.016 | 0.008 | 0.015 |
| 1.2.1-1.2.5 | 5.235 | 0.059 | 0.053 |
| 1.3.1-1.3.2 | 2 | / | / |
| 1.4.1-1.4.2 | 2 | / | / |
| 2.1.1-2.1.6 | 6.101 | 0.02 | 0.016 |
| 2.2.1-2.2.2 | 2 | / | / |
| 2.3.1-2.3.2 | 2 | / | / |
| 3.1.1-3.1.3 | 3.007 | 0.003 | 0.006 |
| 3.2.1-3.2.4 | 4.055 | 0.018 | 0.021 |
